# Glycosyltransferases EXTL2 and EXTL3 cellular balance dictates heparan sulfate biosynthesis and shapes gastric cancer cell motility and invasion

**DOI:** 10.1016/j.jbc.2022.102546

**Published:** 2022-09-28

**Authors:** Catarina Marques, Juliana Poças, Catarina Gomes, Isabel Faria-Ramos, Celso A. Reis, Romain R. Vivès, Ana Magalhães

**Affiliations:** 1 i3S - Instituto de Investigação e Inovação em Saúde, Universidade do Porto, Porto, Portugal; 2 IPATIMUP - Instituto de Patologia e Imunologia Molecular da Universidade do Porto, Porto, Portugal; 3 Programa Doutoral em Biologia Molecular e Celular (MCbiology), Instituto de Ciências Biomédicas Abel Salazar (ICBAS), Universidade do Porto, Porto, Portugal; 4 ICBAS - Instituto de Ciências Biomédicas Abel Salazar, Universidade do Porto, Porto, Portugal; 5 FMUP - Faculdade de Medicina da Universidade do Porto, Porto, Portugal; 6 Univ. Grenoble Alpes, CNRS, CEA, IBS, Grenoble, France

**Keywords:** cancer cell invasion, chondroitin sulfate, exostosin like 2 glycosyltransferase, exostosin like 3 glycosyltransferase, gastric cancer, glycosaminoglycans, heparan sulfate, BSA, bovine serum albumin, CHO, Chinese hamster ovary, CS, chondroitin sulfate, DS, dermatan sulfate, ECM, extracellular matrix, FBS, fetal bovine serum, GAG, glycosaminoglycan, HS, heparan sulfate, HSPG, HS proteoglycan, TBS, Tris-buffered saline, WB, Western blot

## Abstract

Heparan sulfate (HS) proteoglycans (HSPGs) are abundant glycoconjugates in cells’ glycocalyx and extracellular matrix. By acting as scaffolds for protein–protein interactions, HSPGs modulate extracellular ligand gradients, cell signaling networks, and cell–extracellular matrix crosstalk. Aberrant expression of HSPGs and enzymes involved in HSPG biosynthesis and processing has been reported in tumors, with impact in cancer cell behavior and tumor microenvironment properties. However, the roles of specific glycosyltransferases in the deregulated biosynthesis of HSPGs are not fully understood. In this study, we established glycoengineered gastric cancer cell models lacking either exostosin-like glycosyltransferase 2 (EXTL2) or EXTL3 and revealed their regulatory roles in both HS and chondroitin sulfate (CS) biosynthesis and structural features. We showed that EXTL3 is key for initiating the synthesis of HS chains in detriment of CS biosynthesis, intervening in the fine-tuned balance of the HS/CS ratio in cells, while EXTL2 functions as a negative regulator of HS biosynthesis, with impact over the glycoproteome of gastric cancer cells. We demonstrated that KO of *EXTL2* enhanced HS levels along with concomitant upregulation of Syndecan-4, which is a major cell surface carrier of HS. This aberrant HS expression profile promoted a more aggressive phenotype, characterized by higher cellular motility and invasion, and impaired activation of Ephrin type-A 4 cell surface receptor tyrosine kinase. Our findings uncover the biosynthetic roles of EXTL2 and EXTL3 in the regulation of cancer cell GAGosylation and proteoglycans expression and unravel the functional consequences of aberrant HS/CS balance in cellular malignant features.

Heparan sulfate (HS) proteoglycans (HSPGs) comprise an abundant class of glycoconjugates, composed by a core protein with covalently attached HS glycosaminoglycan (GAG) chains. These molecules are ubiquitously expressed, both at the cell surface and in the extracellular matrix (ECM), and play key roles in cellular physiology, impacting cellular proliferation, adhesion and motility, membrane trafficking, formation of extracellular gradients, morphogenesis, and angiogenesis (1). HSPGs are also key players in pathological scenarios, being described as important maestros of cancer cell interaction with the ECM, regulating cancer cell communication, and modulating the tumor microenvironment with impact in disease progression (2). HS chains present high binding affinity to multiple biologically active partners, including transmembrane receptors, ECM structural proteins, and soluble molecules. Therefore, HS chain glycan composition dictates the biological activities of HSPGs (3, 4, 5).

HS chains are linear unbranched polysaccharides composed by repeating disaccharide units of glucosamine and uronic acid residues (6). HS biosynthesis occurs in the Golgi apparatus or at the endoplasmic reticulum–Golgi interface and is organized in three major events: (i) GAG-protein linker assembly, (ii) HS chain polymerization, and (iii) structural modifications of the elongated chain (7). The linker assembly is initiated by the addition of a xylose (Xyl) residue to specific serine (Ser) residues on the protein core (8). This is followed by the sequential addition of two galactose (Gal) and one glucuronic acid (GlcA) residues that form the universal tetrasaccharide GAG-linker GlcAβ1-3Galβ1-3Galβ1-4Xylβ1-*O*-Ser, through which HS chains are covalently attached to the protein moiety (6, 9). The transfer of each monosaccharide residue is catalyzed by a particular glycosyltransferase. The assembly of this GAG-linker also involves additional modification steps, including the transient phosphorylation of the Xyl residue by the kinase FAM20B and phosphatase XYLP, after the addition of the first Gal residue. This step enhances the activity of subsequent acting glycosyltransferases and promotes the maturation of the linker (10, 11, 12). The addition of a single *N*-acetyl glucosamine (GlcNAc) residue to the tetrasaccharide linker then initiates the polymerization of the HS chains, whereas addition of a *N*-acetyl galactosamine (GalNAc) residue would orientate the biosynthesis pathway toward the assembly of chondroitin sulfate (CS)/dermatan sulfate (DS) chains. Addition of this first GlcNAc is catalyzed by two members of the Exostosin (EXT) family, Exostosin-like 2 (EXTL2) and EXTL3, and is followed by further elongation promoted by a hetero-oligomeric complex formed by EXT1 and EXT2, which will catalyze the alternated transfer of GlcNAc and GlcA residues (13, 14, 15, 16). Once polymerized, the pro-heparan chains undergo extensive processing by HS modifying enzymes, including GlcNAc deacetylation and sulfation by *N*-Deacetylase/*N*-Sulfotransferases (NDST1-4) (17), epimerization of GlcA into iduronic acid (IdoA) by Glucuronyl C5-epimerase (18), and sulfation at multiple positions by different *O*-Sulfotransferases (HS2ST1, HS6ST1-3, and HS3ST1-7), leading to the synthesis of mature HS chains displaying highly variable structures and functions (19, 20, 21). These HS chains can then be further modified postsynthetically by heparanase cleavage and 6-*O*-desulfation catalyzed by extracellular 6-*O*-endosulfatases (Sulfs), thus generating additional structural diversity with biological relevance (7).

The members of the Exostosin family that dictate the initiation of HS chain biosynthesis, EXTL2 and EXTL3, have been studied regarding their impact on GAG content using both *in vitro* and *in vivo* models. However, the molecular mechanisms underlying the HS elongation steps are not fully understood yet (22). It has been demonstrated that EXTL3 catalyzes with high efficiency the transfer of the first GlcNAc residue to the mature tetrasaccharide linker and that it can also participate in HS elongation by adding GlcNAc to the growing chain, while being inefficient in the transfer of GlcA (14, 23). Gene KO experiments performed in mouse models revealed that systemic loss of EXTL3 expression led to early embryonic lethality, furthermore the inactivation of *EXTL3* specifically in mice pancreatic islet beta cells caused impaired HS biosynthesis (24), which was also observed in *EXTL3* zebrafish mutants (25). In the same line, more recently, it was shown that KO of *EXTL3* in Chinese hamster ovary (CHO) cells resulted in the abolition of HS expression, which further indicates the crucial role of this enzyme in initiating the biosynthesis of HS chains (26). These results are in agreement with earlier observations that *EXTL3* silencing led to longer HS chains, most probably because of the reduced number of chains being synthesized (27).

There is still reasonable doubt concerning the role of EXTL2 in HS biosynthesis regulation. EXTL2 is an α1,4-N-acetylhexosaminyltransferase, exhibiting dual *in vitro* catalytic activity. Enzymatic assays have shown that this enzyme can act both as a α-GlcNAc and as a α-GalNAc glycosyltransferase toward synthetic linker mimetics. However, it was demonstrated that EXTL2 could not add GlcNAc residues to a mature tetrasaccharide linker substrate (GlcAβ1–3Galβ1–3Galβ1-4Xylβ1-*O*-Ser) (15). More recently, it has been proposed that EXTL2 could mediate an alternative FAM20B-dependent pathway that suppresses HS biosynthesis. According to this model, in the initial steps of HS/CS formation, which entail linker assembly, increased FAM20B kinase activity and/or reduced dephosphorylation by XYLP promote formation and accumulation of the phosphorylated tetrasaccharide linker (GlcAβ1-3Galβ1-3Galβ1-4Xyl(2-*O*-phosphate)). This phosphorylated sequence can be used as substrate by EXTL2 that catalyzes the transfer of a GlcNAc residue and generates an immature phosphorylated pentasaccharide (GlcNAcβ1–4GlcAβ1-3Galβ1-3Galβ1-4Xyl(2-*O*-phosphate)) that cannot be further polymerized by EXT1 and EXT2, thus resulting in the premature termination of HS elongation (12, 28). This model is in agreement with the increment of HS disaccharides observed on CHO *EXTL2* KO cells (26), as well as in *EXTL2*-deficient mice (28, 29). However, the regulatory role played by this enzyme in HS biosynthesis still remains controversial. Whereas the downregulation of EXTL2 resulted in increased HS chains length in human embryonic kidney 293 (HEK293) cells, no significant changes were detected when these cells overexpress EXTL2 (30).

The deregulation of HS biosynthetic machinery has been described as an important event underlying HS abnormal expression in cancer (2). Colorectal (31, 32, 33), breast (34, 35), lung (36), hepatocellular (37), and gastric (38, 39, 40, 41) carcinoma are some of the malignant conditions where altered expression of HSPGs and HS-related genes has been reported. Particularly, gastric tumors have been shown to display aberrant GAGs overall content and altered sulfation patterns (42, 43). However, the mechanisms underlying the alteration of GAG biosynthesis and HS structure in cancer still need to be further clarified. Additionally, the cell- and tissue-specific GAG biosynthesis regulation by GAG-related enzymes, whose expression is variable at the different stages of pathologies, underlines the importance of addressing the impact of these glycosyltransferases’ enzymatic activities on cancer cell behavior and tumor microenvironment remodeling.

In this work, we have evaluated the roles of EXTL2 and EXTL3 in HS biosynthesis in gastric cancer. In addition, we determined the consequences of changes in their expression on the structural features of GAGs in tumor cells’ glycocalyx and their functional impact in cancer cell aggressiveness.

## Results

### EXTL2 and EXTL3 expression regulates the cellular HS content

**Figure 1:**
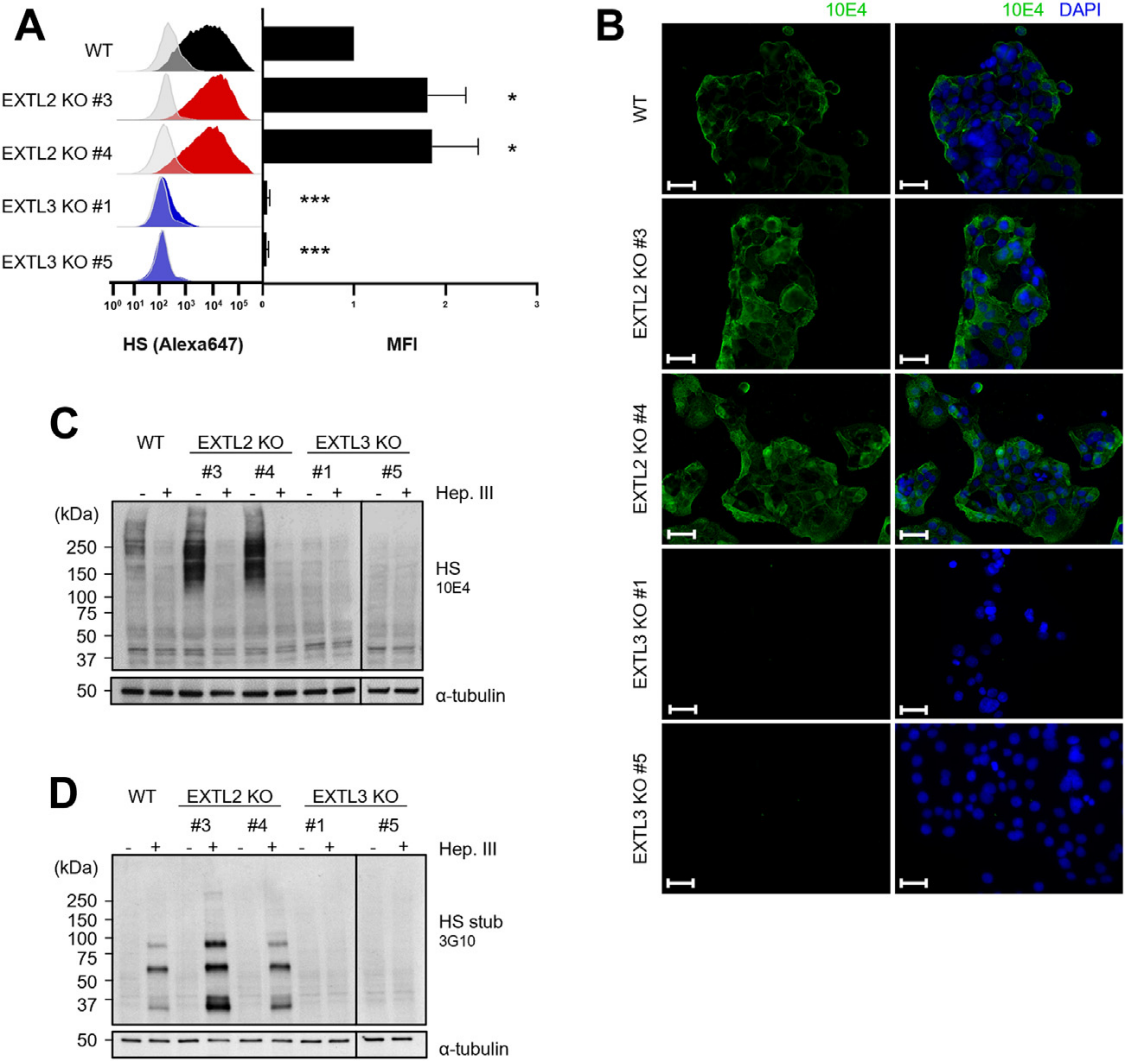
***EXTL2* KO and *EXTL3* KO gastric cancer cells display altered cell surface and overall expression of HS.***A*, the levels of cell surface HS in MKN74 WT, *EXTL2* KO, and *EXTL3* KO cells were quantified by flow cytometry analysis. *Gray* shaded peaks indicate the negative controls. The peaks colored in *solid black*, *red*, or *blue* indicate the labeling with 10E4 mAb of WT, *EXTL2* KO, or *EXTL3* KO cells, respectively. Bar graphs show normalized mean intensity of fluorescence (MIF) + SD. n = 3 independent biological experiments. *B*, immunofluorescence labeling of HS 10E4 epitopes (*green*) was performed in MeOH fixed cells. Nuclei (*blue*) were stained with DAPI. The scale bar represents 50 μm. *C* and *D*, MKN74 WT, *EXTL2* KO and *EXTL3* KO cells were evaluated for HS total content and number of HS chains by WB analyses. Protein lysates were treated with or without Heparinase III (Hep. III) and immunoblotted for undigested HS using the 10E4 mAb (*C*) or HS stubs using the 3G10 mAb (*D*). Images are representative of two independent experiments. DAPI, 4′, 6-diamidino-2-phenylindole; HS, heparan sulfate; WB, Western blot.

To investigate the functional roles of EXTL2 and EXTL3 in HS biosynthesis, KO cell models were generated from the gastric cancer cell line MKN74 *via* CRISPR-Cas 9 genome editing (Fig. S1, *A* and *B*). We first evaluated the effects of *EXTL2* or *EXTL3* gene KO on HS cellular content. Flow cytometry analysis was performed to measure cell surface HS and revealed that in the absence of EXTL2, cells show increased levels of HS (Fig. 1*A*). Immunofluorescence data showed HS staining in cells’ plasma membrane and cytoplasm, for both WT and *EXTL2* KO cells, though a higher number of *EXTL2* KO cells were positive for 10E4 (Fig. 1*B*). In line with these observations, Western blot (WB) assays revealed increased total HS content in *EXTL2* KO cells, as shown by the stronger HS signal covering a wide range of molecular weight (MW), corresponding to the highly variable HS structures decorating HSPGs (Fig. 1*C*). In contrast, KO of *EXTL3* led to the complete depletion of HS, both at the cell surface (Fig. 1*A*) and total cell content (Fig. 1, *B* and *C*). To further investigate the role of EXTL2 and EXTL3 in the regulation of HS biosynthesis, we examined the number of HS chains in our glycoengineered cell models. Cells were treated with Heparinase III (Hep. III) that specifically digests HS chains and generates smaller HS stubs capped with unsaturated GlcA residues, which are recognized by the 3G10 antibody. Protein extracts from *EXTL2* KO cells showed stronger 3G10 labeling, which was particularly evident around 37 kDa (Fig. 1*D*), suggesting an increase in the number of HS chains upon abrogation of EXTL2. *EXTL3* KO cells lacked these HS stub structures (Fig. 1*D*), in accordance with the data shown in Figure 1, *A*–*C*.

### EXTL2 and EXTL3 impact Syndecan-4 expression and its glycosylation profiles

Taking in consideration the impact on HS biosynthesis observed in the glycoengineered cell models and knowing that Syndecans (SDCs) and glycosylphosphatidylinositol-anchored (GPI)–anchored Glypicans (GPCs) are major carriers of HS on epithelial cells, we then evaluated the impact of *EXTL2* and *EXTL3* KO over SDCs and GPCs expression in the gastric cancer cells.

**Figure 2:**
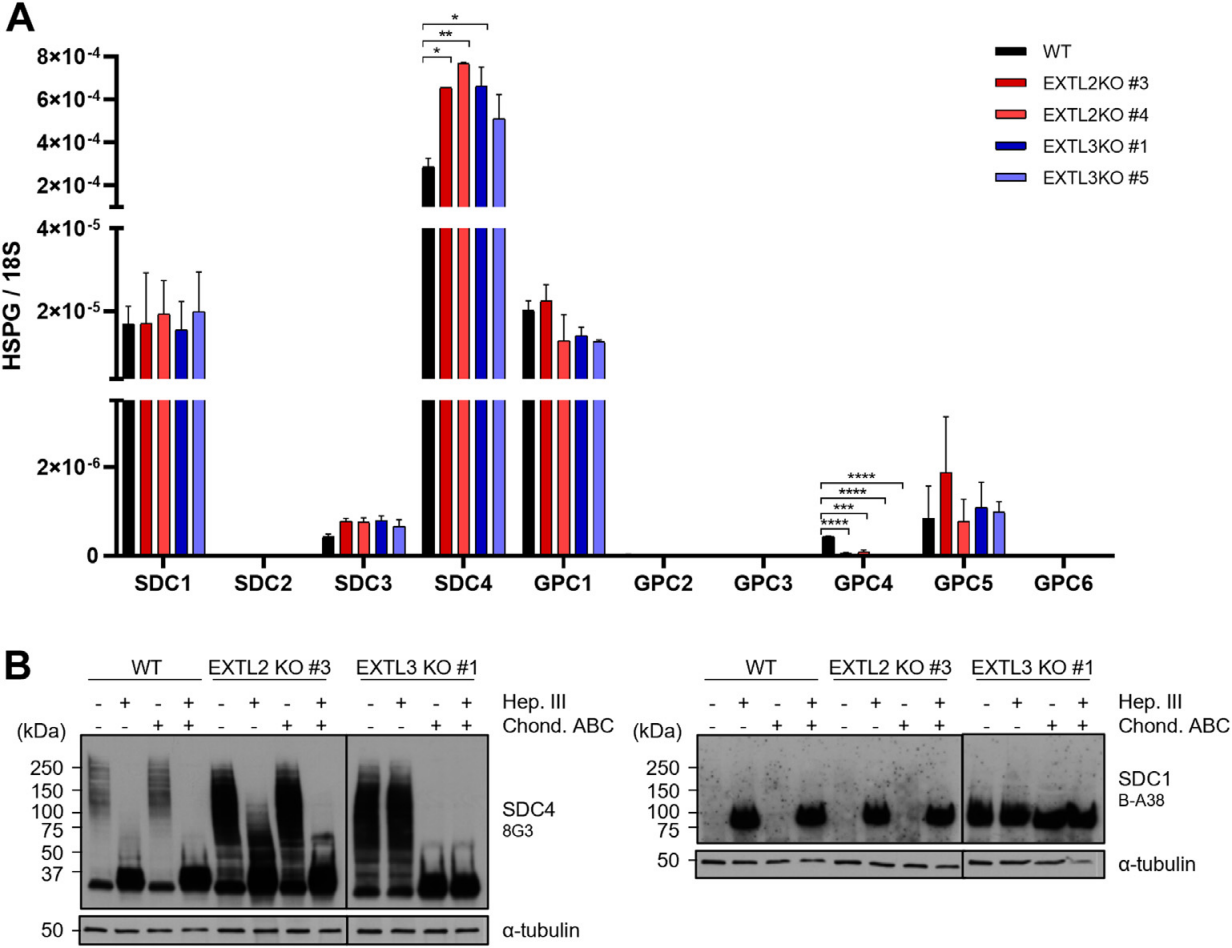
**Modulation of HSPGs’ expression and GAGosylation profiles by *EXTL2* and *EXTL3* KO cells.***A*, the expression of cell surface HSPGs, SDCs 1 to 4, and GPCs 1 to 6 was assessed in MKN74 KO cell models by qRT-PCR. Bar graph shows the mean RQ values for each gene, normalized to the internal control gene 18S + SD. Two independent biological assays with technical triplicates were analyzed. *B*, the protein levels and GAGosylation profiles of SDC4 and SDC1 in *EXTL2* KO and *EXTL3* KO cells were evaluated by WB analysis. Protein lysates were treated with or without Heparinase III (Hep. III) and/or Chondroitinase ABC (Chond. ABC) and immunoblotted for SDC4 and SDC1 using 8G3 and B-A38 mAb, respectively. Images are representative of two independent experiments. HSPG, heparan sulfate proteoglycan; RQ, relative quantification; WB, Western blot.

Transcriptomic analyses revealed that the expression of *GPC1* and *GPC5* was not altered in glycoengineered models, *GPC4* showed lower expression in *EXTL2* KO and *EXTL3* KO, though the expression level in the WT was already very low, and *GPC2*, *GPC3*, and *GPC6* were not detected (Fig. 2*A*). Regarding SDC family members, *SDC1* expression remained unaltered upon KO of *EXTL2* and *EXTL3*, *SDC2* was not detected, and we observed a trend toward *SDC3* overexpression in the glycoengineered models (Fig. 2*A*). Noteworthy, the expression of *SDC4* was significantly increased in both *EXTL2* KO clones, while for *EXTL3* KO, the expression was variable as the increase was only significant for one of the clones (Fig. 2*A*).

Since we observed that MKN74 cells express mainly the SDC1 and SDC4, we further evaluated their protein levels and GAGosylation profiles by WB. Higher SDC4 protein levels were found in the *EXTL2* KO cell model, as evidenced by the increased staining of the fully glycosylated (smear staining) and unglycosylated (37 kDa band staining) SDC4 molecules observed in the nondigested cell lysates (Fig. 2*B*). Cleavage of HS chains by Hep. III on WT cell lysates led to a marked shift in the staining profile of SDC4, from a smear that covered high MW to a sharp staining at 37 kDa (deglycosylated SDC4). In *EXTL2* KO cell lysates upon Hep. III digestion, an intense smear could still be detected in the lower MW range, which was considerably shortened upon combined digestion with Chondroitinase ABC (Chond. ABC) (Fig. 2*B*). These results suggest that in the absence of *EXTL2* gene expression, SDC4 might also be modified with CS/DS chains and that Chond. ABC enzymatic activity is more efficient upon HS removal. This could explain the differences in SDC4 labeling when comparing Hep. III single digestion and Hep. III + Chond. ABC double digestion, as well as the similar staining between nondigestion and Chond. ABC single digestion (Fig. 2*B*). WB analyses of *EXTL3* KO nondigested cell lysates revealed increased signal for glycosylated SDC4, with a large smear between 50 kDa and 250 kDa, while no variations were observed for the labeling of the unglycosylated form at 37 kDa (Fig. 2*B*). This suggests that HS chains might constitute hindrance to the binding of the anti-SDC4 8G3 antibody to the core protein extracellular domain, hence the increased labeling of glycosylated SDC4 in the cell model that lack these chains. No significant variations were detected in the labeling of SDC4 in *EXTL3* KO cells upon single digestion with Hep. III, in agreement with the previous results showing lack of HS in this cell model (Fig. 1). Interestingly, CS/DS cleavage by Chond. ABC eliminated the SDC4 labeling smear (Fig. 2*B*), which further supports that alternative GAGosylation may occur and that the SDC4 expressed in *EXTL3* KO cells is modified with CS/DS chains, instead of HS. Regarding SDC1, HS digestion revealed similar labeling in the WT and *EXTL2* KO cells, which was only detected in Hep. III digested samples, while in *EXTL3* KO cells that lack HS, GAG digestion had no impact over SDC1 labeling profile (Fig. 2*B*).

### EXTL2 and EXTL3 abrogation changes HS and CS GAG structural motifs in gastric cancer cells

To characterize HS and CS glycan structures from *EXTL2* KO and *EXTL3* KO glycoengineered cancer cell lines, we isolated and purified cellular GAGs and performed HS and CS disaccharide analyses using reversed-phase ion pair HPLC (RPIP-HPLC).

**Figure 3:**
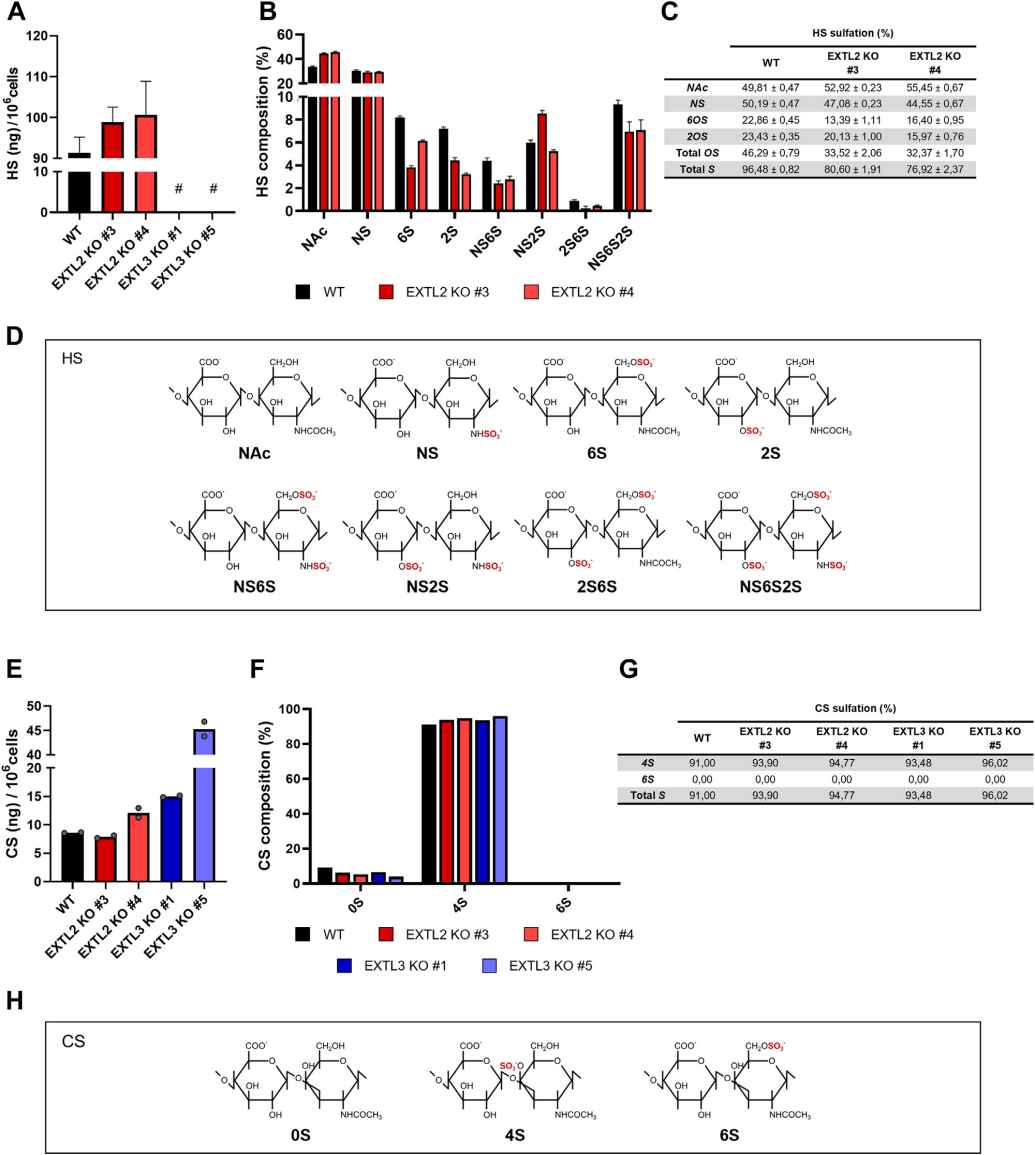
**HS and CS disaccharide composition in *EXTL2* KO and *EXTL3* KO gastric cancer cells.***A*, HS chains isolated and purified from MKN74 cells were digested with a mixture of Heparinases I, II, and III and analyzed by RPIP-HPLC to determine the total amounts and structural composition of HS disaccharides in each cell model. Bar graphs show the mean levels of HS disaccharides, expressed in nanograms, # represents the conditions where HS disaccharides were nondetected. Technical triplicates were analyzed for the WT and *EXTL2* KO models, and technical duplicates were analyzed for *EXTL3* KO models. The analyzed samples reflect pooling from two independent biological replicates. *B*, relative quantities of distinct HS disaccharide units were measured. Bar graph shows the mean levels of each type of HS disaccharide: NAc = ΔHexUA-GlcNAc; NS = ΔHexUA-GlcNS; 6S = ΔHexUA-GlcNAc,6S; 2S = ΔHexUA,2S-GlcNAc; NS6S = ΔHexUA-GlcNS6S; NS2S = ΔHexUA,2S-GlcNS; 2S6S = ΔHexUA,2S-GlcNAc,6S; NS2S6S = ΔHexUA,2S-GlcNS6S. *C*, table represents the mean values of sulfation content. *N*A*c* = *N*-Acetylation; *NS* = *N*-Sulfation; *6S* = 6-*O*-Sulfation; *2S* = 2-*O*-Sulfation; total *OS* = total *O*-Sulfation, total *S* = total Sulfation (*N*- and *O*-sulfation). *D*, illustrative representation of HS disaccharide structures is included. *E*, CS chains isolated and purified from MKN74 cells were digested with Chondroitinase ABC and were also analyzed by RPIP-HPLC. Bar graph shows relative mean quantities of distinct CS disaccharide units. Technical duplicates were analyzed from the pool of two independent biological samples. *F*, relative quantities of distinct CS disaccharide units were measured. Bar graph shows the levels of each type of CS disaccharide: 0S = ΔHexUA-GalNAc 4S = ΔhexUA-GalNAc,4S; 6S = ΔhexUA-GalNAc,6S. One dataset is represented. *G*, table represents the CS sulfation content in each cell model. *4S* = 4-*O*-Sulfation; *6S* = 6-*O*-Sulfation; total S = total Sulfation (4-*O*- and 6-*O*-Sulfation). *H*, illustrative representation of CS disaccharide structures is included. CS, chondroitin sulfate; HS, heparan sulfate; RPIP-HPLC, reversed-phase ion pair HPLC.

Structural analyses showed a trend for increased HS disaccharide content in *EXTL2* KO cells, in comparison with WT, while in *EXTL3* KO cells HS disaccharides were not detected (Fig. 3*A*). Also, HS composition appeared to vary between WT and *EXTL2* KO cells (Fig. 3, *B* and *C*). Results suggest that in the absence of EXTL2 expression, HS exhibits increased levels of nonmodified *N*-acetylated disaccharides, along with a decrease in overall disaccharide *O*-sulfation (Fig. 3, *B*–*D*).

Regarding CS composition, *EXTL3* KO resulted in increased CS disaccharide content (Fig. 3*E*). This observation is in agreement with the high MW smear observed for SDC4 in the *EXTL3* KO clones, which was abolished after Chon. ABC digestion (Fig. 2*B*). The effect of *EXTL2* KO on CS expression was more heterogeneous among clones, with only one presenting increased CS disaccharide units (Fig. 3*E*). Furthermore, no major variations in CS composition were detected for MKN74 glycoengineered cells compared to the WT (Fig. 3, *F*–*H*).

### Aberrant GAGosylation profiles displayed by *EXTL2* KO cancer cells promote motile and invasive phenotypes

GAGs are key modulators of ECM physical and biochemical properties, which are capable of binding to different proteins and allow proteoglycans to act as cell surface coreceptors and mechanosignaling transducers, ultimately impacting cell behavior (44). Taking these biological features in consideration, we resorted to our glycoengineered cell models to disclose the impact of the distinct GAGosylation profiles on the migration and invasion capabilities of gastric cancer cells.

**Figure 4:**
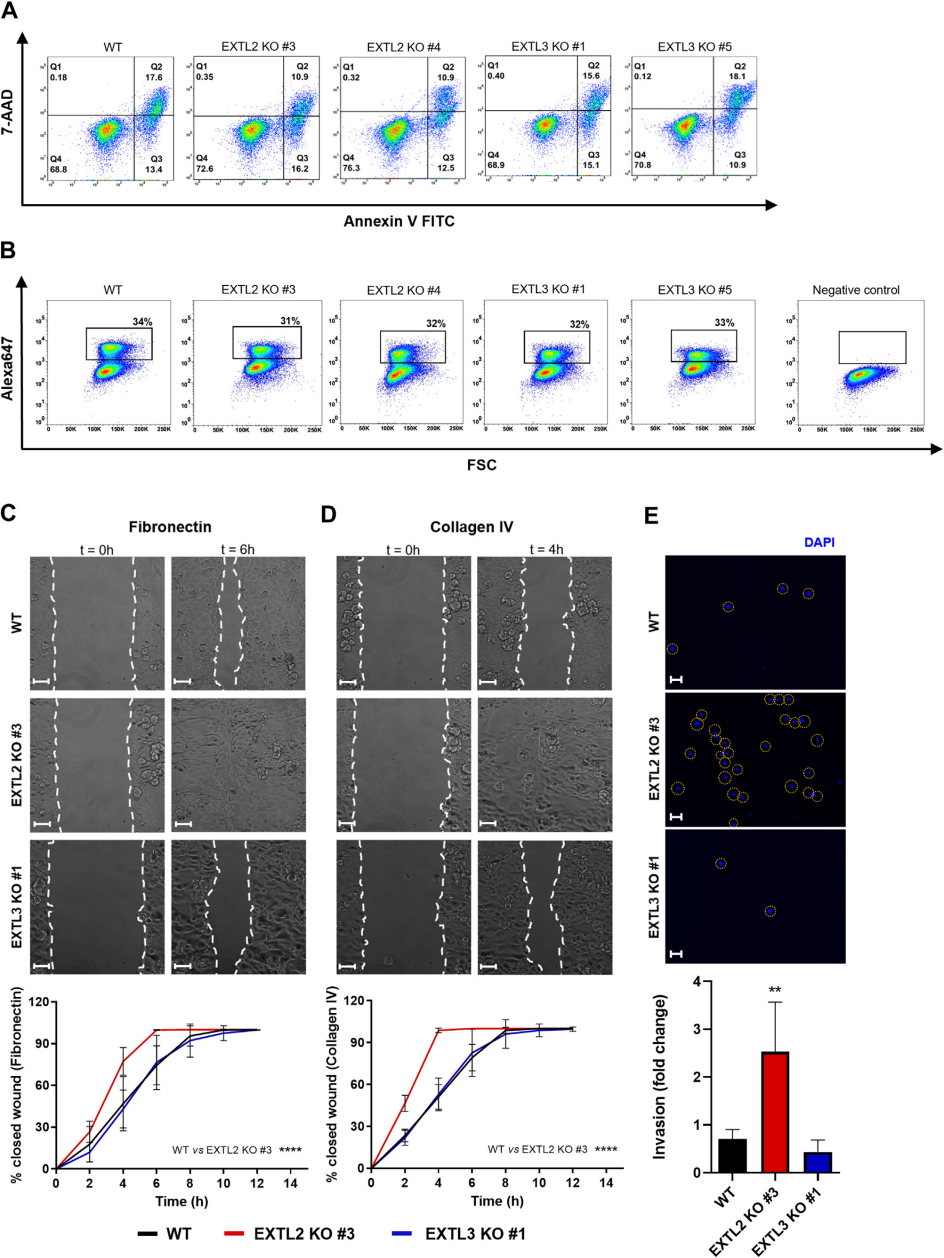
**Impact of EXTL2 and EXTL3 expression in MKN74 gastric cancer cell biological features.***A*, cell viability was evaluated by performing 7-AAD/Annexin V-FITC cell labeling. Dot plots show percentages of viable cells (Q4), cells in early apoptosis (Q3), cells in late apoptosis (Q2), and cells in necrosis (Q1). Data from one representative assay from two independent biological replicates. *B*, cell proliferation was assessed by measuring EdU incorporation in cell’s DNA by flow cytometry. Dot plots show the percentage of proliferating cells as EdU-Alexa647 positive cells, highlighted in the *black box*. A representative dot plot of a negative control (without EdU) is shown. Data from one representative assay from two independent biological replicates. *C* and *D*, migration capabilities of MKN74 WT and one representative clone of the *EXTL2* KO and *EXTL3* KO cell models were addressed by performing a wound healing migration assay. Representative pictures of the wounded cell monolayer in fibronectin (*C*) and collagen IV (*D*) coated slides are depicted together with the graphs showing the mean percentages of closed wounds + SD over time. The scale bar represents 50 μm. Two independent biological experiments with technical triplicates were analyzed. *E*, invasion was evaluated by performing a Matrigel invasion assay. Microscope images show highlighted DAPI stained nuclei of the invasive cells counted for each condition. The scale bar represents 50 μm. Bar graph shows the mean percentage of invading cells + SD. Two independent biological experiments with technical triplicates were analyzed.

We first assessed whether *EXTL2* or *EXTL3* KO would impact cells viability and proliferation and found that abrogation of EXTL2 or EXTL3 had no impact, neither on the percentage of viable cells (Q4, Fig. 4*A*) nor on the cells’ proliferating percentage (Fig. 4*B*).

To determine the functional impact of altered GAGosylation in cancer cell motility, we performed wound healing assays and evaluated the migration rates of *EXTL2* KO and *EXTL3* KO cells on fibronectin (Fig. 4*C*) and collagen IV (Fig. 4*D*) coated surfaces. Interestingly, the *EXTL2* KO cells presented higher migration rates in both coatings, while no significant differences were observed for *EXTL3* KO cells when compared to the WT (Fig. 4, *C* and *D*). All cells migrated faster in the presence of collagen IV than in fibronectin.

In order to assess if *EXTL2* KO would also impact cancer cell invasion, we evaluated cells’ ability to break down and penetrate matrigel-coated membranes, which is a basement membrane–like matrix, enriched in ECM proteins. Remarkably, *EXTL2* KO cells displayed significantly higher invasion capabilities, while *EXTL3* KO cells showed no differences compared to the WT (Fig. 4*E*). These results indicate that *EXTL2* KO cells’ aberrant display of GAGs promotes higher aggressiveness features such as more motile and invasive phenotypes, which are not dependent on altered viability or proliferation.

### GAGs remodeling by *EXTL2* KO impacts ephrin type-A receptor 4 activation

Receptors tyrosine kinase (RTKs) activation and downstream signaling pathways are known to promote aberrant cellular events related to the acquisition of cancer hallmark capabilities, which contribute to tumor progression. GAG chains, in particular HS, are able to modulate cancer cell signaling by coupling multiple biologically active ligands, like growth factors, to their targeted RTKs. This prompts HSPGs to act as important scaffolds for protein–protein interactions and to trigger receptors activation and subsequent signaling transduction (45).

**Figure 5:**
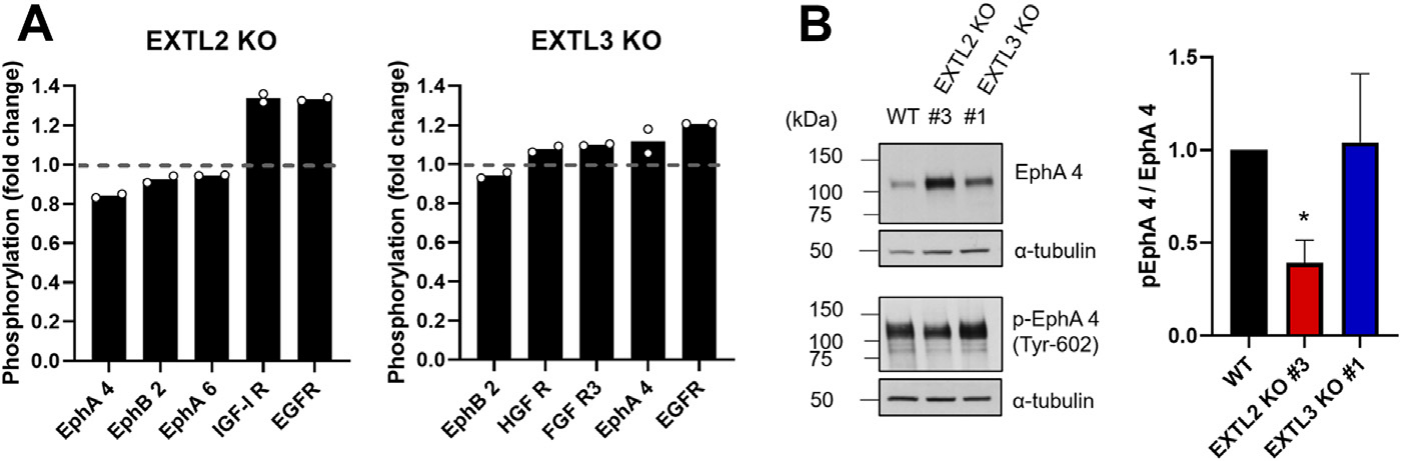
**Abrogation of EXTL2 expression decreases EphA 4 phosphorylation in MKN74 gastric cancer cells.***A*, phospho-RTKs in MKN74 glycoengineered cell models were analyzed. Bar graphs show the mean relative phosphorylation of different RTKs expressed in *EXTL2* KO and *EXTL3* KO cells normalized to WT values (WT = 1). For each representative KO clone (*EXTL2* KO #3 and *EXTL3* KO #1), only RTKs whose phosphorylation fold change varied over 0.05 compared to the WT are shown. *B*, the activation of EphA 4 was measured by WB analysis. α-Tubulin was used as loading control. Bar graph shows the mean values of the normalized phosphorylated receptor + SD. n = 3 independent biological experiments. WB, Western blot.

To uncover the impact of specific GAGosylation profiles from *EXTL2* KO and *EXTL3* KO gastric cancer cells in the activation of RTKs, we screened the phosphorylation state of multiple RTKs using a phospho array (Fig. S2, *A* and *B*). Results from the phospho-RTK array indicated that *EXTL2* KO cells exhibited higher insulin-like growth factor 1 receptor (IGF-IR) and epidermal growth factor receptor (EGFR) activation and lower Ephrin type-A receptor 4 (EphA 4) phosphorylation when compared to the WT. Interestingly, *EXTL3* KO cells showed increased EGFR and IGF-IR phosphorylation, similar to *EXTL2* KO, but higher EphA 4 activation (Fig. 5*A*). Overall, the RTK-array revealed EphA 4 as an interesting candidate to be further studied since *EXTL2* KO and *EXTL3* KO cells presented an opposite trend in terms of its activation.

Validation analysis by WB for total EphA 4 receptor amounts and phosphorylation at Tyr-602 revealed that abolishing the expression of EXTL2 in gastric cancer cells, which is concomitant with higher HS levels, resulted in increased levels of the total receptor with a statistically significant reduction on its activation (Fig. 5*B*). This result supports a possible role of HS remodeling by *EXTL2* KO in cellular signaling networks mediated by EphA 4. The validation analysis of EphA 4 phosphorylation at Tyr-602 in *EXTL3* KO cells did not show statistically significant differences in comparison with WT cells (Fig. 5*B*). Regarding EGFR activation, we analyzed by WB the specific phosphorylation of Tyr-1068 and Tyr-1086 in the different clones and could not confirm increased phosphorylation at these particular Tyr residues (Fig. S2*C*).

## Discussion

HSPGs are important components of cells’ glycocalyx and ECM. By binding to multiple biological ligands *via* their sulfated HS chains, HSPGs modulate cancer cells’ interactions with ECM and signaling networks, ultimately controlling tumor microenvironment and disease progression (2). In the present study, we disclosed the regulatory roles of EXTL2 and EXTL3 in GAG and HSPG biosynthetic pathways, in the context of gastric cancer, and evaluated the impact of the resulting altered GAGosylation in cancer cells’ motility and signaling features.

For this purpose, we have established glycoengineered gastric cancer cell models by knocking out either *EXTL2* or *EXTL3* from MKN74 cells and evaluated the impact of both glycosyltransferase KOs over GAG biosynthesis. *EXTL2* KO led to increased cell levels of HS, while *EXTL3* KO fully abolished HS expression (Figs. 1 and 3*A*). These data demonstrate the role of EXTL2 as a negative regulator of HS biosynthesis and further corroborate EXTL3-mediated promotion of HS polymerization. These results are in agreement with previous observations in different cellular (26) and animal (24, 25, 28, 29) models. Interestingly, a previous report has revealed that HS biosynthesis could be driven by EXTL2 and EXT2 in the context of EXT1-deficient mouse L fibroblasts, whereas in the same model, EXTL3 silencing had little effect on cellular HS levels (46). Together with our data, these observations suggest that the phosphorylated pentasaccharide linker, formed after the transfer of a GlcNAc residue by EXTL2 to the phosphorylated tetrasaccharide, could serve as substrate for EXT2, whereas usually it is not further polymerized by the heterodimeric complex EXT1-EXT2 (28). In contrast, complete abrogation of HS biosynthesis upon KO of *Ext1* and/or *Ext2* was reported in CHO cells (26). Overall, these results support that these glycosyltransferases act in a tissue- and cell-specific manner. Therefore, it remains critical to further study the complex interactions of GAG biosynthetic machinery elements and its impact on the regulatory mechanisms underlying GAG biosynthesis in different cellular contexts and disease.

We investigated the impact of deregulated HS biosynthesis in the expression and GAGosylation features of two major HS carriers, SDC1 and SDC4. Our data showed that SDC4 was significantly overexpressed in *EXTL2* KO cells (Fig. 2, *A* and *B*), which indicates that HS chains, beside impacting HSPG cellular functions, might also intervene in the regulation of cell membrane HSPG expression and therefore in the remodeling of cell glycoproteome. The enhanced SDC4 expression could also explain the increased number of HS chains detected for this particular cell model (Fig. 1*D*). Analysis of protein levels also showed increased SDC4 detection on *EXTL3* KO cells (Fig. 2*B*), which was not reflected at the mRNA levels for both clones (Fig. 2*A*). Therefore, we hypothesize that 8G3 antibody has higher binding affinity to its target epitopes in HS-free SDC4 expressed in *EXTL3* KO cells. This may also explain the higher signal intensity observed for the 8G3-labeled de-GAGosylated SDC4 in the WT. Regarding *EXTL2* KO cells, our data suggest that SDC4 overexpression and consequent higher levels of available 8G3 binding epitopes overcome the effects of HS hindrance (Fig. 2*B*). It has been previously reported that the endoglycosidase heparanase influences the cellular distribution of SDC1 and SDC4 and plays a role in HSPG turnover (47). In the same study, not only HSPG core protein but also HS chains were found to be critical for the efficient HSPG-heparanase binding and its cellular uptake, whereas other GAGs like CS could not perform this task (47). Therefore, we can also hypothesize that the aberrant GAGosylation profile of SDC4, resulting from *EXTL3* KO, might impact internalization, degradation, and/or recycling of this HSPG, which could ultimately lead to its abnormal cellular accumulation.

Regarding HSPG GAGosylation profiles, GAG enzymatic digestion assays revealed that SDC4, that normally carries only HS, was abnormally modified with CS chains in the absence of EXTL3 expression (Fig. 2*B*), which was further corroborated by CS structural analyses that showed increased CS disaccharide levels in *EXTL3* KO cell models (Fig. 3*E*). Curiously, Gopal *et al.* have previously shown that deregulation of HS biosynthesis by mutagenesis of SDC4 GAG attachment sites affected HS/CS ratio with increased CS levels (48). Furthermore, accumulation of CS was also reported in CHO cell mutants exhibiting impaired biosynthesis of HS chains (26, 49). In line with these observations, significant higher levels of CS were found on *extl3* mutant zebrafish larvae, and a larger proportion of CS chains were specifically attached to SDC4 upon silencing of *EXTL3* in HEK293 (50). Our results support that abrogation of EXTL3 favors the accumulation of available tetrasaccharide linker substrates, attached to specific HSPGs, which cannot be polymerized by EXT1 and EXT2 and are therefore available for priming CS assembly by chondroitin N-acetylgalactosaminyltransferases. However, the rationale for HSPG glycosylation profile changes seen in *EXTL2* KO cell models (Fig. 2*B*) might be different. Izumikawa T. *et al.* have shown that after linker assembly, the CS polymerase chondroitin N-acetylgalactosaminyltransferase-1 adds preferably a GalNAc residue to the extremity of phosphorylated tetrasaccharide linker, generating the pentasaccharide GalNAcβ1-4GlcAβ1-3Galβ1-3Galβ1-4Xyl(2-*O*-phosphate). This reaction is accompanied by the dephosphorylation of the linker by XYLP and followed by CS elongation catalyzed by chondroitin (Chn) polymerases (51). Similarly, we hypothesize that in our *EXTL2* KO cell models, lack of EXTL2 expression in MKN74 gastric cancer cells could lead to the accumulation of phosphorylated tetrasaccharides that are not capped with GlcNAc residues and are thus available to undergo this particular CS biosynthesis pathway. At the same time, EXTL3-driven HS biosynthesis could be conserved (Fig. 2*B*). Moreover, the changes observed in the labeling profile of SDC4 upon double digestion with Hep. III + Chond. ABC, when compared with single digestion with Hep. III, further support modification of SDC4 with CS in *EXTL2* KO cell models (Fig. 2*B*). Overall, these results suggest the existence of a complex regulatory interplay between CS and HS biosynthesis, supporting the hypothesis that the balance between HS and CS enzymatic machinery determines cellular HS/CS ratio (50). Interestingly, HS glycosyltransferases that act in the initial steps of the chain elongation, like EXTL3, appear to have a preponderant activity in this balance.

Additionally, we addressed the effects of altered GAGosylation over tumor cells motility and signaling events. Cellular functional analysis showed that *EXTL2* KO and concomitant HS increase and SDC4 overexpression promoted a more motile and invasive phenotype in MKN74 gastric cells (Fig. 4, *C*–*E*). Previous reports have depicted the functional role of HS and HSPGs in modulating adhesion and migration in different cell lines (52, 53). SDC4, for example, displays important roles in motility as a key component of cells’ focal adhesions. In line with our results, Gopal S. *et al.* revealed a direct correlation between HS chains and the role of SDC4 in mechanotransduction, showing that the expression of SDC4 modified with multiple HS chains is required for correct organization of cells’ cytoskeleton actin components and formation of focal adhesions (48).

GPCs have also been previously associated to changes in tumor cell migration and invasion in various types of cancer (54, 55). GAG chains, HS in particular, were shown to be significantly important to the roles displayed by GPCs in cancer progression (56). Our data revealed that only *GPC1*, *GPC4*, and *GPC5* were expressed in MKN74 gastric cancer cells. *GPC4* was downregulated in both KO models (Fig. 2*A*), therefore it is unlikely that it plays a role in promoting a more aggressive phenotype. In addition, no changes were observed on *GPC1* and *GPC5* upon KO of *EXTL2* (Fig. 2*A*). Still, these transcriptomic data does not allow to exclude that altered GPCs’ glycosylation features might contribute to the observed phenotype.

Notably, we observed that *EXTL3* KO cells presented unaltered migration and invasion (Fig. 4, *C*–*E*). Since functional overlap between CS and HS has been previously described in different cell events, we speculate that increased CS content may compensate the absence of HS and re-establish *EXTL3* KO cell motility capabilities (57). Further studies are warranted to understand if CS acts on similar HS-governed signaling pathways or activates distinct signaling cascades that ultimately lead to similar functional behavior.

HS binds with high affinity to several biologically active proteins and contributes to the role of coreceptor displayed by many HSPGs by modulating ligand interactions with their targeted cell surface receptor. Particularly, HS can tether RTK ligands and promote the receptors activation and downstream signaling pathways (58, 59). Therefore, we have investigated the role of the specific HS profiles displayed by *EXTL2* KO and *EXTL3* KO cells in RTKs’ activation. Interestingly, our results showed that abrogation of EXTL2 and subsequent cellular HS increase resulted in decreased EphA 4 activation in gastric cancer cells (Fig. 5). EphA 4 belongs to a large and unique family of erythropoietin-producing hepatocellular (Eph) receptors, whose members have been shown to modulate cell morphology, adhesion, migration, and invasion (60). EphA 4 activation has been previously associated with increased migration of cancer cells (61, 62), and particularly in gastric cancer, EphA 4 upregulation has been reported as a bad prognostic factor (63, 64, 65). However, the *EXTL2* KO model that displays a high motility phenotype shows decreased phosphorylation of this receptor (Fig. 5). The analysis of the interaction of heparin and HS with ephrin ligands and Eph receptors showed that only ephrin-A3 ligands bind to both, while EphA 4 receptor, which was only tested for heparin interactions, exhibited no detectable direct binding. In addition, it was reported that ephrinA3-HS binding was essential to mediate EphA 4 activation (66). HS sulfation is known to determine HS binding affinity and impact HS biological roles (67, 68). Therefore, we cannot exclude that the changes in HS sulfation detected in *EXTL2* KO (Fig. 3*B*) might contribute to impair HS-ligand and/or HS-receptor interactions and interfere with activation and that an alternative pathway might underlie the *EXTL2* KO motility features. Additional analyses are warranted to further understand the molecular determinants underlying HS-mediated EphA 4 activation and downstream regulation.

**Figure 6:**
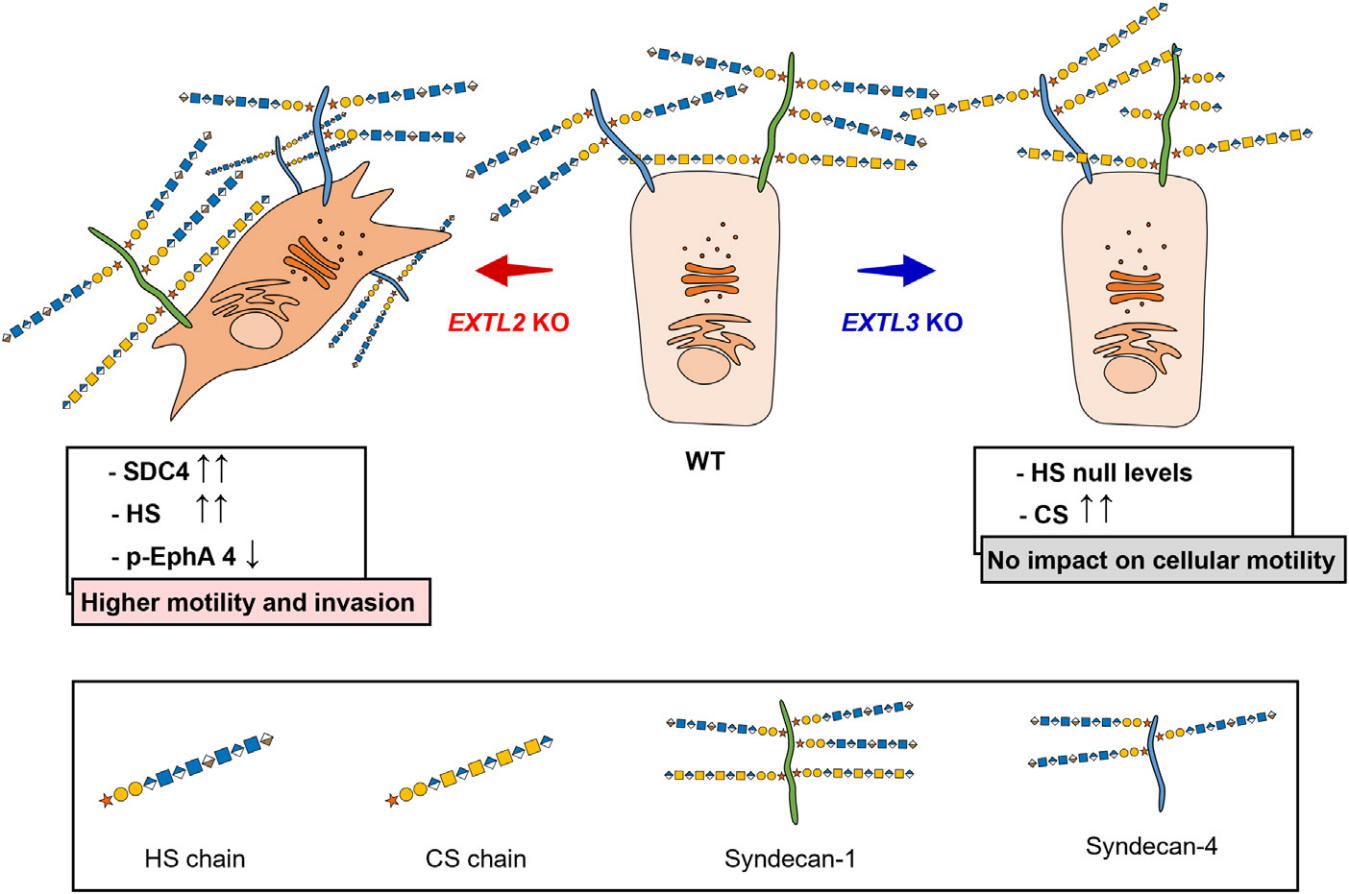
**Schematic representation depicting the impact of EXTL2 or EXTL3 abrogation on gastric cancer cells’ glycocalyx remodeling and functional features.** GAG biosynthesis is regulated by EXTL2 and EXTL3. KO of *EXTL2* promoted HS overproduction and SDC4 overexpression in gastric cancer cells, ultimately inducing increased migration and invasion capabilities concomitant with lower EphA 4 activation. On the other hand, EXTL3 elimination triggered CS biosynthesis, while blocking HS biosynthetic pathways, and induced the expression of abnormal SDC4 molecules modified with CS chains, instead of HS, which might contribute to rescue cell motility properties. Glycan structures are represented according to the Symbol Nomenclature for Glycans (SNFG) format (75). CS, chondroitin sulfate; GAG, glycosaminoglycan; HS, heparan sulfate.

As illustrated in Figure 6, our data revealed that lack of EXTL2 enzymatic activity results in the remodeling of GAGs on cell glycocalyx, promotes SDC4 upregulation and overproduction of HS with altered sulfation profile. Ultimately, these glycosylation changes were shown to promote a highly motile and invasive phenotype and to downregulate the activation of EphA 4 cell surface receptor. On the other hand, *EXTL3* KO blocks HS synthesis and triggers CS biosynthesis pathways. Our results showed that the absence of EXTL3 catalytic activity leads to the expression of SDC4 molecules abnormally modified with CS chains, which might compensate for the lack of HS and rescue cell functional behavior.

In conclusion, we showed that the abrogation of the tumor suppressor gene *EXTL2* in gastric cancer cells contributes to aggressive cellular features, such as increased motility and invasion. This further supports the importance of the molecular mechanisms underlying dysregulation of HS biosynthetic and postsynthetic modification pathways in cancer. Currently, there is very limited knowledge regarding the expression and impact of EXTL2 and EXTL3 in the context of cancer pathologies (69, 70, 71). Evaluating the expression profiles of these enzymes in clinical samples of gastric cancer patients at different stages of the disease could be of high clinical value as prognostic factors for patient’s stratification or novel targets for therapy.

## Experimental procedures

### Cell lines, cell culture, and genetic engineering

In this study, we resorted to the human gastric adenocarcinoma cell line MKN74, obtained from the Japanese Collection of Research Bioresources Cell Bank. Cells were cultured in RPMI1640 (Gibco) culture medium, supplemented with 10% (v/v) fetal bovine serum (FBS) (Biowest), at 37 °C, under 5% (v/v) CO_2_ conditions.

The glycoengineered *EXTL2* KO and *EXTL3* KO cell models were generated from the gastric cancer cell line MKN74 *via* CRISPR-Cas 9 genome editing. Briefly, cells were cotransfected with Cas9 endonuclease vector containing GFP and a plasmid with a validated guide RNA for the target gene (Table 1). KO clones were obtained by single cell sorting of fluorescence-activated single cell sorting enriched nuclease-expressing cells and gene KO was validated by indel detection using amplicon and restriction fragment length polymorphism combination analysis, as previously described (72). Two clones were selected for each target gene and gene KO confirmed by Sanger sequencing and indels were validated through Tracking of Indels by Decomposition (TIDE) methodology (Fig. S1, *A* and *B*) (73).

**Table 1:** List of validated gRNAs used to target *EXTL2* and *EXTL3* based on (76) and results of indel analysis

Gene	gRNA sequence	Target exon	Indels	
*EXTL2*	CAGCTACCAGTAATAATACG	1/4	clone 3	clone 4
			+1	+1
*EXTL3*	GGTGGGGAACGAGCTGTGCG	1/5	clone 1	clone 5
			+1	+1

Abbreviations: gRNA, guide RNA.

### Antibodies

The antibodies and conditions used for the analysis described later are listed in Table 2.

**Table 2:** List of antibodies and conditions used for WB, immunofluorescence, and flow cytometry (FACS) assays

Antibodies	Applications and dilutions			Source/Reference
	WB	IF	FACS	
HS (F58-10E4)	1:600	1:200	1:600	Amsbio
HS stub region (F69-3G10)	1:500	1:100		Amsbio
SDC1 (B-A38)	1:125			Abcam
SDC4 (8G3)	1:2000			(77)
EphA 4 (4C8H5)	1:250			ThermoFisher
pEphA 4 [Tyr602] (EP2731)	1:1000			bioNova científica s.l.
EGFR (D38B1)	1:1000			Cell Signaling Technology
pEGFR [Tyr1068] (D75A)	1:1000			Cell Signaling Technology
pEGFR [Tyr1086] (36-9700)	1:2000			Invitrogen
α-Tubulin (DM1A)	1:10000			Sigma–Aldrich
Horseradish Peroxidase-conjugated α mouse IgG	1:5000			Jackson ImmunoResearch Laboratories, Inc
Horseradish Peroxidase-conjugated α mouse IgG1	1:8000			Jackson ImmunoResearch Laboratories, Inc
Horseradish Peroxidase-conjugated α mouse IgM	1:5000			Jackson ImmunoResearch Laboratories, Inc
Rabbit α-mouse Ig – FITC		1:70		DAKO
Alexa Fluor® 647 α mouse IgM			1:400	Jackson ImmunoResearch Laboratories, Inc

Abbreviations: FACS, fluorescence-activated cell sorting; IF, immunofluorescence; WB, Western blot.

### HS detection by flow cytometry assay

Cells previously cultured in 75 cm^2^ flasks were detached with versene (Gibco), resuspended in fresh medium, and centrifuged at 300*g* for 5 min. Cells (1 × 10^6^ cells/ml) were washed two times in PBS and 1% bovine serum albumin (BSA) (Sigma–Aldrich) solution, and each wash followed by centrifugation at 300*g* for 5 min. The cell pellet was resuspended and incubated 1 h at room temperature (RT) with the primary antibody 10E4 (Table 2) in PBS and then washed again with PBS 1% BSA solution and incubated with APC fluorochrome-conjugate antimouse IgM. For negative controls, cell pellets were incubated with APC fluorochrome-conjugate antimouse IgM only. Finally, cells were washed again and resuspended in PBS 1% BSA solution and filtered for analysis. Data were acquired using FACscan Canto II and analyzed with the FlowJo software (v10; BD Biosciences). Mean intensity of fluorescence values measured for the KO clones were normalized to the mean intensity of fluorescence values of WT, which were defined as a unit value. Three independent experiments were analyzed.

### Fluorescence microscopy

About 70% to 80% confluent cells seeded and grown on 12-well microscope glass slides (IBIDI) were washed twice with PBS and fixed on ice for 10 min with methanol (Thermo Fisher Scientific). Cells were washed again with PBS, blocked with rabbit serum (Dako), diluted in a ratio of 1:5 in PBS 10% BSA, and incubated with the primary antibody 10E4 (Table 2) in PBS 5% BSA, overnight at 4 °C. This was followed by incubation with rabbit α mouse Ig–FITC conjugated secondary antibody for 30 min, at RT. Finally, cells were incubated with 100 μg/ml 4′, 6-diamidino-2-phenylindole diluted 1:100 in PBS, and mounted in VectaShield mounting medium (Vector Laboratories). Immunofluorescence analysis for HS stubs was also performed on methanol fixed cells. After fixation, cells were permeabilized by incubation with 0.5% Triton X-100 in PBS for 20 min. Cells were washed with PBS and then incubated with 0.2 M NH_4_Cl in PBS for 20 min. For HS digestion, cells were treated with Heparinase III (EC 4.2.2.8; 5 mU/ml) (Amsbio) in Tris-buffered saline (TBS) 0.1% BSA, supplemented with calcium acetate (0.1 mM), both diluted in 1:200 in water, for 2 h at 37 °C. Cells were washed again with PBS, blocked with rabbit serum (Dako), diluted in a ratio of 1:5 in PBS 10% BSA, and incubated with the primary antibody 3G10 (Table 2) in PBS 5% BSA, overnight at 4 °C. The following labeling steps were performed as previously described for 10E4 staining. Microscope images were obtained with the Zeiss Axio Imager Z1, Axiocam MR ver3.0, and Axiovision 4.8 Software (Carl Zeiss).

### WB

Total protein lysates were obtained from confluent cells scraped and lysed with lysis buffer 17 (R&D Systems) supplemented with 1 mM sodium orthovanadate (Sigma–Aldrich), 1 mM PMSF (Sigma–Aldrich), and cOmplete protease inhibitor cocktail (Roche). The protein concentration of these lysates was determined using the DC protein assay (Bio-Rad). Total protein lysates (30 μg for HS, 12.5 μg for HS stub, 25 μg for SDC1, SCD4, and EGFR, 35 μg for EphA 4 and 20 μg for p-EphA 4) were incubated with 4× Laemmli sample buffer (Bio-Rad), supplemented with 10% β-mercaptoethanol, at 98 °C for 5 min. The protein samples were loaded and run in 4% to 15% precast polyacrylamide gels (Bio-Rad) and blotted to a nitrocellulose membrane (GE Healthcare Life Sciences). The membranes were blocked for 1 h at RT, with TBS 5% BSA and 0.05% Tween 20 (Sigma–Aldrich) (TBS-T 0.05%) buffer, and then incubated overnight at 4 °C with primary antibodies (Table 2) diluted in the blocking solution. Membranes were washed with TBS-T 0.05% and incubated for 1 h at RT with horseradish peroxidase–conjugated secondary antibodies diluted in the blocking solution. New washes with TBS-T 0.05% were performed, membranes were developed, and the protein bands visualized with ECL chemiluminescent WB detection reagent and films (GE Healthcare Life Sciences). For WB analysis, immunoblot band densities related to the expression and phosphorylation of EphA 4 and EGFR receptors in each KO clone were normalized to the band density values of the WT. Receptor’s phosphorylation levels were normalized to the levels of the total receptor. Results are represented as the mean values of the normalized phosphorylated receptor + SD. Three independent experiments were analyzed.

### Glycosaminoglycan enzymatic digestion assay

HS enzymatic digestion was performed by incubating 250 μg of protein lysates of each sample with Heparinase III (EC 4.2.2.8; 5 mU/ml) (Amsbio) in TBS 0.1% BSA, supplemented with calcium acetate (0.1 mM), overnight, at 37 °C with continuous mixing. Twenty-five micrograms of the resulting digested samples were then analyzed through WB analysis, by labeling HS digested resulting stubs with 3G10 antibody (Table 2). CS enzymatic digestion was performed by incubating 50 μg of protein lysates of each sample with Chondroitinase ABC (EC 4.2.2.4; 10 mU/μl) (Amsbio) in TBS 0.1% BSA, supplemented with 50 mM Tris–HCl pH 7.5, 50 mM NaCl, 2 mM CaCl_2_, overnight, at 37 °C with continuous mixing.

### Real-time PCR analysis

For mRNA quantification/gene expression analysis, total RNA was extracted from MKN74 WT and glycoengineered cell models (*EXTL2* KO and *EXTL3* KO) using TRIzol Reagent (ABP Biosciences). Five micrograms of RNA from each cell condition was converted into complementary DNA *via* reverse transcription by incubation with random hexamers/primers using the SuperScript IV Reverse Transcriptase Kit (Invitrogen). Real-time PCR was performed using for each condition, per well 4 μl of complementary DNA diluted 1:10 in ultrapure water, 0.6 μl of each primer at 10 μM (Table 3), 10 μl of PowerUp SYBR Green Master Mix (Thermo Fischer Scientific), and ultrapure water up to a final volume of 20 μl. The real-time PCR run was performed using a 7500 Fast Real-Time PCR System (Applied Biosystems). Relative quantification values were determined for each gene and 18S ribosomal RNA was used as a housekeeping gene to normalize relative gene expression (Relative quantification = 2^-(target Ct – 18S Ct)^). Two independent experiments with triplicates were analyzed.

**Table 3:** List of primers used in the real-time PCR analysis

Gene	Primer Fw	Primer Rv
*SDC1*	5′-ATGGCTCTGGGGATGACTCT-3′	5′-GTGGGAATAGCCGTCAGGAG-3′
*SDC2*	5′-AGGATGTAGAGAGTCCAGAGCT-3′	5′-TGTATCCTCTTCGGCTGGGT-3′
*SDC3*	5′-TGACATCCCTGAGAGGAGCA-3′	5′-GCTACCACCTCATTGGCTGT-3′
*SDC4*	5′-CCGGAGCCCTACCAGACGAT-3′	5′-AGGCACCAAGGGATGGACAA-3′
*GPC1*	5′-CATCGGGTGTGGAGAGTG-3′	5′-TGAGCGTGTCCCTGTTGTC-3′
*GPC2*	5′-CTGGGACACGACCTGGAC-3′	5′-GCCATCCAGTCATCTGCATAC-3′
*GPC3*	5′-CTGCTTCAGTCTGCAAGTATGG-3′	5′-GTGGAGTCAGGCTTGGGTAG -3′
*GPC4*	5′-AGTGTGGTCAGCGAACAGTG-3′	5′-CAAACATATCATTCAGGGATTTCTC-3′
*GPC5*	5′-GCCGCCCTGTAAGAACAC-3′	5′-TCATTCCATGCTTCTCTTTGC-3′
*GPC6*	5′-CCAGGCATAAGAAATTTGACG-3′	5′-CATGTACAGCATGCCATAGGTC-3′
*18S*	5′-CGCCGCTAGAGGTGAAATTC-3′	5′-CATTCTTGGCAAATGCTTTCG-3′

### GAG purification and HS/CS disaccharide analysis

To evaluate HS disaccharide composition, GAGs were isolated and purified, and structural analysis was performed as described in (74). For preparation and purification of GAG chains, cells were exhaustively digested with trypsin (45 min at 37 °C). Cellular debris was discarded by centrifugation and supernatants were recovered and incubated with 250 U/μl Benzonase (Merck) and 2 mM MgCl_2_ for 2 h at 37 °C. Benzonase was then inactivated by heating supernatants at 96 °C for 5 min and additional centrifugation was performed to discard nucleic acids. The recovered supernatants were applied to a DEAE-Sephacel column (Pharmacia Biotech) equilibrated in 20 mM phosphate pH 6.5. The column was then extensively washed with 20 mM phosphate pH 6.5, 0.3 M NaCl, and then GAGs were eluted with 20 mM phosphate pH 6.5, 1 M NaCl. Samples were desalted over a Pd-10 column (GE Healthcare), lyophilized, and stored at −20 °C prior to analysis. For HS disaccharide analysis, samples were resuspended in 100 mM sodium acetate, 0.5 mM calcium acetate, pH 7.1, and then digested into disaccharides by incubation with a mix of heparinase I, II, and III (10 mU each) for 48 h at 37 °C. Compositional analysis was performed by reversed-phase ion pair HPLC, by applying samples to a C18 reversed phase column equilibrated in a H_2_O/acetonitrile (8.5%) buffer supplemented with 1.2 mM of ion-pairing tetra-*N*-butylammonium hydrogen sulfate, then resolved using a multistep NaCl gradient calibrated with HS disaccharide standards. On-line post-column disaccharide derivatization was achieved by the addition of 2-cyanoacetamide (0.25%) in NaOH (0.5%), followed by fluorescence detection (excitation 346 nm, emission 410 nm). For CS disaccharide analysis, purified samples were digested into disaccharides by incubation with 500 mU of Chondroitinase ABC in 50 mM Tris–HCl pH 7.5, 50 mM NaCl, 2 mM CaCl_2_, for 24 h at 37 °C. The following compositional analysis was performed similarly to what was previously described for HS disaccharide analysis using CS disaccharide standards instead.

### Annexin V viability assay

To evaluate cell viability, 1.5 × 10^5^ cells per well were seeded on 6-well plates (Corning Incorporated Costar). After 48 h in culture, cells were trypsinized (Biowest), resuspended on the medium they were cultured in, and centrifuged at 300*g* for 5 min. The supernatant was discarded and the cell pellet was resuspended again in fresh medium. Cells were washed twice in PBS, followed by another two washes with Annexin V binding buffer (BioLegend). The resulting cell pellet was resuspended and incubated with Annexin V-FITC (BioLegend) diluted with a 1:40 ratio in Annexin V binding buffer for 15 min at RT. Cells were filtered and data were acquired using a BD FACSCanto II (Becton, Dickinson and Company) and analyzed with the FlowJo Software (v10). Two independent biological replicates were analyzed.

### Proliferation assay

Cell proliferation was determined using Click-It Plus EdU Alexa Fluor 647 Flow Cytometry Assay Kit (Molecular Probes, Invitrogen) using the BD FACSCanto II flow cytometer. Cells were counted and adjusted to 2.5 × 10^5^ cells/ml and seeded in a 25 cm^2^ flask in RPMI supplemented with 10% FBS and left to grow at 37 °C in 5% CO_2_ atmosphere conditions. In parallel, cells were also grown in simple media (without FBS). Forty-eight hours later, cells were incubated with 10 μM of EdU for 1 h 30 min prior to harvesting. EdU nontreated cells served as control, while cells in simple media served as a cell arrest control. Detection of EdU incorporation into DNA was performed according to the manufacturer’s instructions. Briefly, harvested cells (as described earlier) were washed in PBS 1% BSA and fixed in 100 μl Click-It1 fixative for 15 min at RT in the dark. Cells were then washed again in PBS 1% BSA and resuspended in 100 μl saponin-based permeabilization and wash reagent. Click-It1 EdU reaction cocktail was prepared according to the manufacturer’s instructions and added. Samples were incubated for 30 min at RT in the dark and washed with saponin-based permeabilization and wash reagent. Cell pellet was resuspended in 300 μl saponin-based permeabilization and wash reagent, acquired using BD FACSCanto II cytometer, and analyzed using FlowJo software (v10). Two independent biological replicates were analyzed.

### Wound healing assay

About 5.3 × 10^5^ cells/ml were seeded in each side of a silicon insert, previously adhered to a well of a μ-Slide 8 Well Collagen IV or Fibronectin coated (IBIDI). Cells were kept in culture for 24 h at 37 °C in 5% CO_2_ atmosphere conditions. The inserts were then removed, cells were washed with RPMI1640 culture medium supplemented with 10% FBS, and fresh supplemented medium was added to each well. Time-lapse microscopy was performed using Leica DMI6000 (Wetzlar) and three bright field images per well/condition were acquired for 24 h with intervals of 10 min. The wound healing rate was evaluated by measuring the total wound area at each time point using the ImageJ software (https://imagej.nih.gov/ij/). Migration assay results were depicted as the average values of the percentage of closing wound + SD. The percentage of closing wound was calculated by subtracting the area of the open wound at the first time point (t = 0 h) to the area determined for each time point, followed by normalization of the resulting values to the wound area determined for the first time point (t = 0). Two independent biological experiments with at least technical triplicates were analyzed.

### Matrigel invasion assay

Invasion assays were performed resorting to a 24-well plate of BD BioCoat Matrigel Invasion Chambers (BD Biosciences). About 2 × 10^5^ cells were initially seeded and incubated in RPMI1640 medium in the upper chamber for 24 h at 37 °C in 5% CO_2_ atmospheric conditions. The lower side of the well contained only RPMI1640 medium supplemented with 10% FBS. The matrigel-coated chambers were then washed with PBS, the noninvasive cells adhered on the inner side of the chamber were removed with a cotton swab, and the chambers were fixed in ice-cold methanol for 10 min and air dried. The chambers were washed with PBS, the matrigel-coated membranes were removed and mounted in glass coverslips with VectaShield mounting medium with 4′, 6-diamidino-2-phenylindole (Vector Laboratories). Microscope images of the stained nuclei were obtained resorting to the Zeiss Axio Imager Z1, Axiocam MR ver3.0, and Axiovision 4.8 Software (Carl Zeiss) and the total number of invasive nuclei was counted. Invasion assay results were represented as the average values of the fold changes of the number of invasive cells + SD. The number of invasive cells of the KO models was normalized to WT, which was defined as a unit value. Two independent biological experiments with technical triplicates were analyzed.

### RTK phosphorylation array

The activation state of important RTKs was determined by using the Proteome Profiler Human Phospho-RTK Array Kits (R&D Systems #ARY001B) following the manufacturer’s instructions. Briefly, confluent cells (1 × 10^7^ cells/ml) were solubilized in lysis buffer 17 (R&D Systems) supplemented with cOmplete protease inhibitor cocktail (Roche) and PhosSTOP phosphatase inhibitor (Sigma–Aldrich). The protein concentration of these lysates was determined using the DC protein assay (Bio-Rad). The RTK array membranes were incubated with the Array buffer 1 for 1 h at RT with continuous shacking. Three hundred micrograms of whole cell lysates were diluted in Array buffer 1 to a final volume of 1.5 ml and incubated on the RTK array membranes overnight at 4 °C with continuous shaking. Membranes were then washed thrice with 1× wash buffer, 10 min each wash, followed by incubation with 1:5000 antiphospho tyrosine-horseradish peroxidase detection antibody diluted in Array buffer 2 for 2 h at RT with continuous shaking. Membranes were washed again thrice with 1× wash buffer and finally were developed and visualized with ECL chemiluminescent detection reagent and films. For dot quantification, densitometry was evaluated and the phosphorylation fold change of each RTK was calculated for each KO cell model by comparison with the RTK phosphorylation values determined for the WT. The following calculation was performed: fold change = 1 – (average WT p-RTK densitometry/average KO p-RTK densitometry).

## Statistical analysis

Statistical analysis was carried out using GraphPad Prism 6 (GraphPad Software Inc) and statistical significance was considered when *p* values were ≤0.05 (∗ means *p* ≤ 0.05; ∗∗ means *p* ≤ 0.01; ∗∗∗ means *p* ≤ 0.001; ∗∗∗∗ means *p* ≤ 0.0001). For flow cytometry, invasion assay and WB analysis, statistical significance of WT *versus EXTL2* KO and WT *versus EXTL3* KO was determined using unpaired Student’s *t* test with Welch’s correction, with a 95% interval of confidence. For real-time PCR statistical significance was determined with one-way ANOVA using Tukey’s test for multiple comparisons. Migration assay statistical significance was calculated by two-way ANOVA with a 95% interval of confidence and shown for each KO cell model relative to the WT.

## Data availability

All data described and discussed are contained within the article.

## Supporting information

This article contains supporting information.

## Conflict of interest

The authors declare that they have no conflicts of interest with the contents of this article.
