# Supplementary material for: Glycosyltransferases EXTL2 and EXTL3 cellular balance dictates heparan sulfate biosynthesis and shapes gastric cancer cell motility and invasion
Source: J Biol Chem. 2022 Sep 28;298(11):102546. doi: 10.1016/j.jbc.2022.102546 (PMC9637574; doi:10.1016/j.jbc.2022.102546)
Supplement: Supporting information [file mmc1.pdf]

Supporting Information

Glycosyltransferases EXTL2 and EXTL3 cellular balance dictates Heparan Sulfate biosynthesis and shapes gastric cancer cell motility and invasion

Catarina Marques, Juliana Poças, Catarina Gomes, Isabel Faria-Ramos, Celso A. Reis,

Romain R. Vivès, Ana Magalhães

Contents

1. Figure S1. Validation of the glycoengineered *EXTL2* KO and *EXTL3* KO cell models generated via CRISPR-Cas 9 genome editing.

2. Figure S2. Assessment of RTKs phosphorylation state in MKN74 *EXTL2* KO and *EXTL3* KO cancer cell models.

Figure S1.

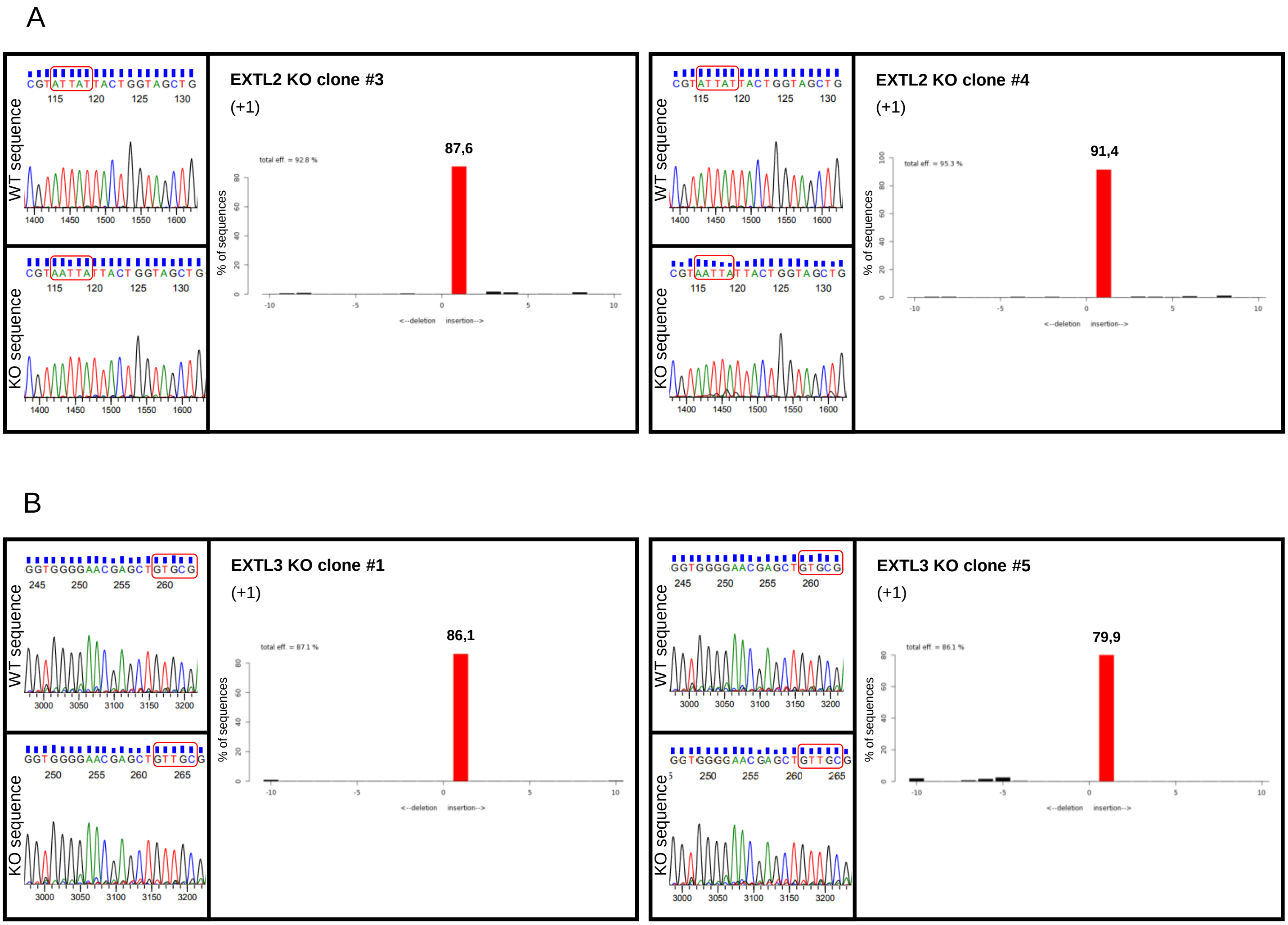

Figure S1. Validation of the glycoengineered *EXTL2* KO and *EXTL3* KO cell models generated via CRISPR-Cas 9 genome editing. KO validation through Sanger sequencing for **(A)** *EXTL2* KO clones #3 and #4 and **(B)** *EXTL3* KO clones #1 and #5. Indel sequencing was validated through Tracking of Indels by Decomposition (TIDE) methodology. The MKN74 WT sequence (top left panel) was used as the reference (control) for comparison with all KO clones.

Figure S2.

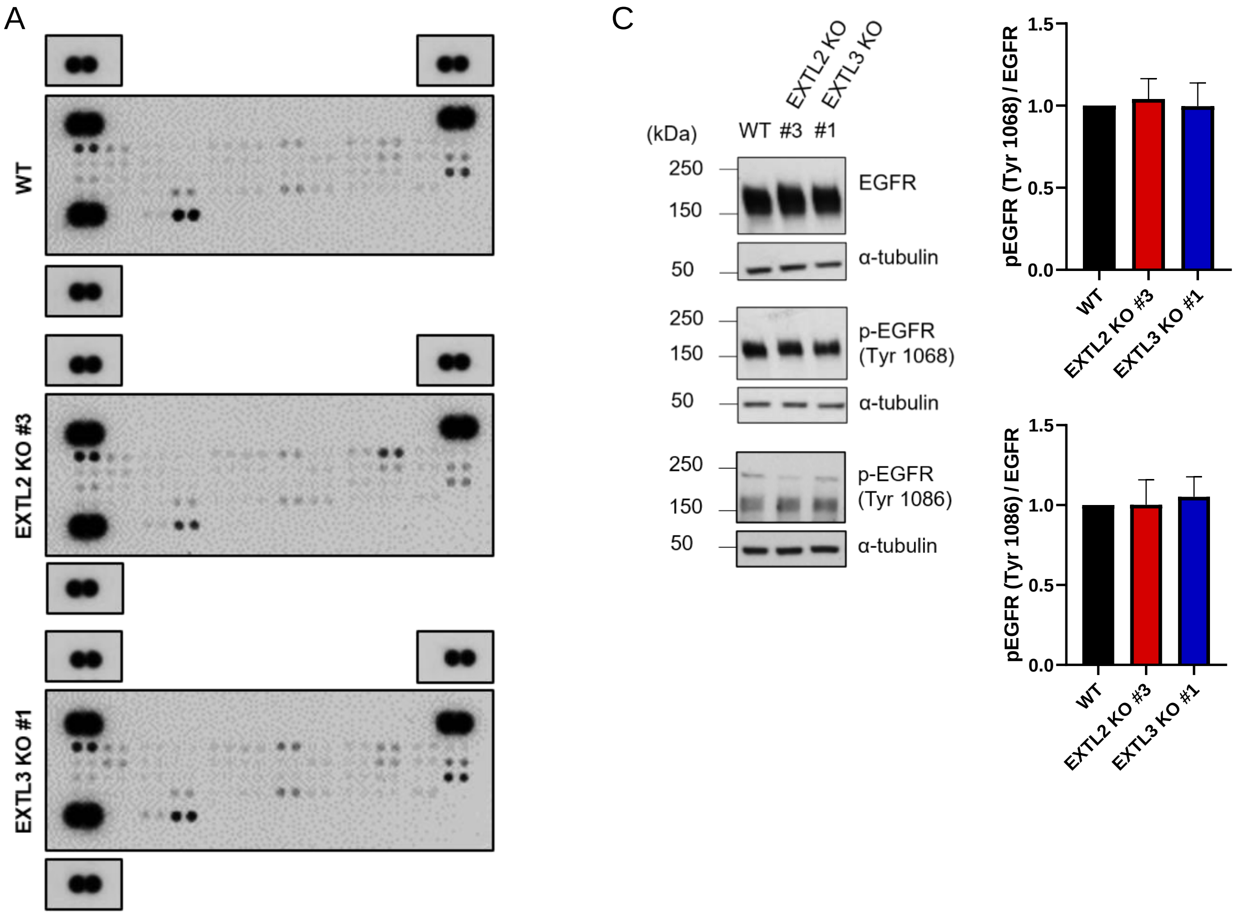

B

	1	2	3	4	5	6	7	8	9	10	11	12	13	14	15	16	17	18	19	20	21	22	23	24
A	Reference spot																						Reference spot	
B	EGFR		ErbB2		ErbB3		ErbB4		FGF R1		FGF R2α		FGF R3		FGF R4		Insulin R		IGF-I R		Axl		Dtk	
C	Mer		HGF R		MSP R		PDFG Rα		PDFG Rβ		SCF R		Flt-3		M-CSF R		c-Ret		ROR1		ROR 2		Tie-1	
D	Tie-2		TrkA		TrkB		TrkC		VEGF R1		VEGF R2		VEGF R3		MuSK		EphA 1		EphA 2		EphA 3		EphA 4	
E	EphA 6		EphA 7		EphB 1		EphB 2		EphB 4		EphB 6		ALK		DDR 1		DDR 2		EphA 5		EphA 10			
F	Reference spot				EphB 3		RYK																Control (-) PBS	

Figure S2. Assessment of RTKs phosphorylation state in MKN74 *EXTL2* KO and *EXTL3* KO cancer cell models. (A) Evaluation of the phosphorylation state of 49 RTKs expressed in MKN74 WT, *EXTL2* KO and *EXTL3* KO cells. Each couple of dots represents the staining intensity for each p-RTK. Less exposed images of the reference spots are shown above and below each blot. **(B)** Schematic illustration showing the location of each RTK specific antibody in the human p-RTK array. **(C)** The activation of EGFR was measured by WB analysis using antibodies that specifically recognize EGFR Tyr-1068 and Tyr1086. α-Tubulin was used as loading control. Band density values related to EGFR expression and phosphorylation were normalized to the band density values of the WT, which were defined as one unit. p-EGFR levels were normalized to the levels of the total receptor. Bar graph shows the mean values of the normalized phosphorylated receptor + SD. n=3 independent biological assays.
